# Population structure of the Indonesian giant tiger shrimp *Penaeus monodon*: a window into evolutionary similarities between paralogous mitochondrial DNA sequences and their genomes

**DOI:** 10.1002/ece3.1616

**Published:** 2015-08-06

**Authors:** Muslihudeen A Abdul-Aziz, Gerhard Schöfl, Grit Mrotzek, Haryanti Haryanti, Ketut Sugama, Hans Peter Saluz

**Affiliations:** 1 Leibniz Institute for Natural Product Research and Infection BiologyBeutenbergstr. 11a, 07745, Jena, Germany; 2 Friedrich Schiller University JenaJena, Germany; 3 DKMS Life Science Lab GmbHFiedlerstr. 34, 01277, Dresden, Germany; 4 Institute for Mariculture Research and Development – IMRAD, Ds PenyabanganBr. Gondol PO. Box 140, Singaraja, Bali, 81101, Indonesia; 5 Research and Development Center for AquacultureJL Ragunan 20, Pasar Minggu, Jakarta Selatan, 12540, Indonesia

**Keywords:** Microsatellite, mtCR, mtDNA, Numts, paralogous sequences, *Penaeus monodon*

## Abstract

Here we used both microsatellites and mtCR (mitochondrial DNA control region) sequences as genetic markers to examine the genetic diversity and population structure of *Penaeus monodon* shrimp from six Indonesian regions. The microsatellite data showed that shrimp from the Indian and the Pacific Ocean were genetically distinct from each other. It has been reported previously that *P. monodon* mtCR sequences from the Indo-Pacific group into two major paralogous clades of unclear origin. Here we show that the population structure inferred from mtCR sequences matches the microsatellite-based population structure for one of these clades. This is consistent with the notion that this mtCR clade shares evolutionary history with nuclear DNA and may thus represent nuclear mitochondrial pseudogenes (Numts).

## Introduction

**Figure 1:**
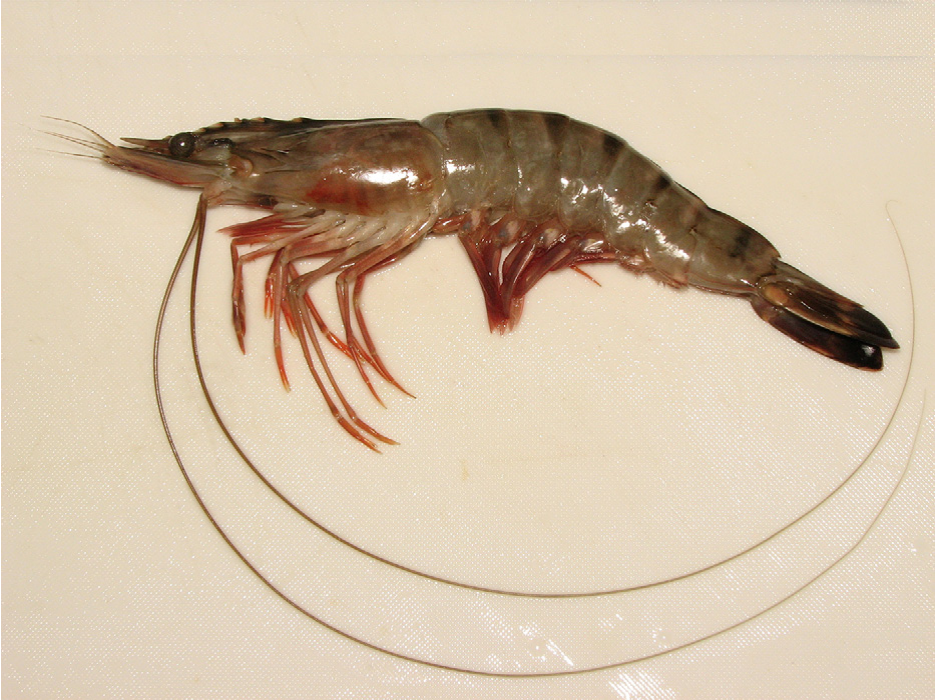
*Penaeus monodon* (black tiger shrimp).

*Penaeus monodon* (black tiger shrimp) (Fig.1) is a marine crustacean widely cultured in the Indo-Pacific, especially in the Indonesian coastal region (Holthuis 1976). During the past two decades, the dramatic growth in mariculture industry has propelled Indonesia into one of the top producers of shrimp worldwide (FAO (Food & Agriculture Organisation), 2014). As a result, wild shrimp populations have become liable to devastation, as most fry are sourced from the wild due to their superior fertility and fecundity (Rosenberry 2004; Gillett 2008). In order to improve the output of local farmers as well as to understand the effect of this exponential growth of production of *P. monodon* in Indonesian waters, it is necessary to better understand the structure and migration patterns of wild shrimp populations in these waters.

Studies carried out during the last two decades aimed at better understanding the underlying population structures, phylogenetic relationships, and geographic distributions of various marine organisms inhabiting the Indonesian coastal region have consistently found this region to be a major center of marine biodiversity (Klinbunga et al. 1998; Benzie et al. 2002; Sugama et al. 2002). While a number of historical biogeographic factors have been proposed to explain the diversity in this region, the factor that is most widely accepted is the complete closure of the Indian and Pacific oceans during the ice ages (Palumbi 1997).

A number of approaches using a variety of molecular tools such as allozymes (Sugama et al. 2002), mtDNA-RFLP (Brooker et al. 2000), or sequencing the mtCR (mtDNA control region) (Walther et al. 2011) have been used to study the population structure of *P. monodon*. Interestingly, Walther et al. (2011) inferred the presence of paralogous nuclear copies of mitochondrial origin (Numts) in addition to the putatively true mitochondrial sequences in a large number of *P. monodon* samples, concluding that this may have misled inferences of population structure in *P. monodon* in the past.

Here, we aim to further explore the genetic diversity and population structure of *P. monodon* in Indonesian waters by combining microsatellite and mtCR data, specifically taking into account the presence of nuclear copies of mtCR.

## Materials and Methods

### Sample collection and DNA extraction

**Figure 2:**
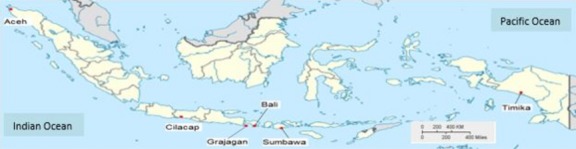
Map indicating the six sample collection sites in Indonesian waters.

A total of 115 wild *P. monodon* samples were collected from 6 geographic locations across the Indonesian coastal region: Aceh (*n *=* *27), Bali (*n *=* *17), Cilacap – Central Java (*n *=* *11), Grajagan – East Java (*n *=* *20), Sumbawa – West Nusa Tenggara (*n *=* *20), and Timika – Papua (*n *=* *20) (Fig.2). All samples were obtained from wild populations. Pleopods and muscle tissue were preserved in 95% ethanol. DNA was extracted from muscle tissue (c. 50–100 mg) using Bio&Sell DNA Mini Kit (Bio&Sell e.K, Feucht, Germany) as described in the kit instruction manual and stored at −20°C for further analyses.

### Amplification and analyses of microsatellite markers

According to Pan et al.'s (2004) study, a set of 15 polymorphic markers (Table2) which resulted in high expected heterozygosity values (*H*_E_ >0.92) were chosen for genotyping.

All PCR experiments were performed in 96-well plates using an Applied Biosystem Veriti 96-well fast Thermal Cycler (Carlsbad, CA, USA). Each reaction (20 *μ*L) contained 10 ng DNA, 10 *μ*L of 2x QIAGEN Fast Cycling PCR Master Mix (Venlo, Limburg, Netherlands), and 0.02 *μ*mol/L of both, forward and reverse primers.

The thermocycling profile consisted of an initial activation step of 3 min at 95°C, followed by 40 cycles of denaturation for 10 sec at 95°C, annealing for 30 sec at 50–60°C (according to primers used) and extension period of 2 min at 72°C. PCR products were visualized on high-resolution synthetic polymer-based matrix, Spreadex EL 400–600 gels (Elchrom Scientific, Cham, Switzerland) run in an Elchrom ORIGINS apparatus and sized with M3 size marker (Elchrom Scientific). Staining was performed with 1xGelStar nucleic acid gel stain (Lonza, Basel, Switzerland). For confirmation, samples with ambiguous genotypes were independently amplified and scored twice.

### Data analysis

Based on microsatellite data (Jarne and Lagoda 1996), FSTAT version 2.9 (Goudet 1995) was used to describe the intrapopulation genetic diversity using the number of alleles (*A*), the number of private alleles per loci (*A*_p_), the allelic richness based on sample size (*A*_r_), the expected heterozygosity (*H*_E_) in comparison with the observed heterozygosity (*H*_O_) (Saitou and Nei 1987), and the inbreeding coefficient (*F*_IS_) for the six shared microsatellite loci that were examined.

Null alleles were mitigated using FreeNA (Chapuis and Estoup 2007). To test whether the observed genotype frequencies of individual loci deviated from the expected HWE (Hardy–Weinberg equilibrium) within a population, and to test for heterozygote deficiency across loci and populations, GENEPOP v 4.0 (Raymond and Rousset 1995) was used.

To investigate interpopulation structures, the genetic distance (pairwise *F*_ST_ value) between any two populations was calculated by comparing allele frequencies (Wright 1949). FSTAT v.2.9 (Goudet 1995) was used to determine the statistical significance of an *F*_ST_ value by performing 10,000 permutations. A matrix of *F*_ST_ values was then used to visually display the relationships between populations. The resulting matrices were also used to construct phylogenetic trees using the neighbor-joining (NJ) method as implemented in QuickTree (Howe et al. 2002).

To determine the most likely sources of genetic ancestry (*K*) without considering sampling locations, a Bayesian clustering analysis was performed using the admixture model of ancestry and correlated allele frequency parameters in STRUCTURE v2.3 (Pritchard et al. 2000) with 1.2 × 10^6^ MCMC iterations (2 × 10^5^ of which were discarded as burn in). In order to obtain the real number of sources of genetic ancestry, the modal value of Δ*K*, a quantity based on the second-order rate of change with respect to *K* of the likelihood function was used (Evanno et al. 2005).

Tested *K* values ranged from 1 to 8, and for each value of *K*, six replicates were performed. The most likely *K* was determined by plotting their posterior probabilities. Comparing posterior probabilities predicted for each *K* value, the value immediately below the posterior probability values with the smallest differential was taken as the most likely number of sources of genetic ancestry.

### Sequence analyses of mtDNA control gene region

mtDNA control region sequences for the 115 *P. monodon* samples have been determined previously (Walther et al. 2011). Of these 115 individuals, 111 had mtCR sequences that matched the putatively authentic mitochondrial sequences (haplogroup A; fig. 1 in Walther et al. 2011). Twenty-six of these 111 individuals had additional *mtDNA*-control-region-like sequences, a majority of which belonged to haplogroup C (fig. 1 in Walther et al. 2011; 24 individuals). Haplogroup A comprised of *P. monodon* from Aceh (*n *=* *25), Bali (*n *=* *17), Cilacap (*n *=* *11), Grajagan (*n *=* *20), Sumbawa (*n *=* *19), and Timika (*n *=* *19), while haplogroup C consisted of shrimp from Aceh (*n *=* *8), Bali (*n *=* *8), Cilacap (*n *=* *2), Grajagan (*n *=* *3), and Sumbawa (*n *=* *3). Haplogroup C had no samples from Timika as the haplogroup was found to be present specifically only in *P. monodon* from the western edge of the Indo-Pacific (Walther et al. 2011).

For our analysis, sequences from haplogroups A and C were analyzed. These sequences were obtained post-editing and FaBox v1.41 (Villesen 2007) was used to collapse the sets of sequences into haplotypes and convert them into input files for Arlequin v3.5 (Excoffier and Lischer 2010) which was used to calculate the pairwise *F*_ST_ matrices. The resulting matrices were then used to construct phylogenetic trees using the neighbor-joining (NJ) method utilizing the QuickTree software (Howe et al. 2002). Sequences were deposited at GenBank (accession numbers –HQ724473).

### Comparative analysis for the elucidation of the likely source of the paralogous sequences

To elucidate the most likely source of the paralogous sequences found (haplogroup C) in addition to the putatively authentic mitochondrial sequences (haplogroup A), for this analysis only shrimp individuals were retained for which both, microsatellite data and mtCR, data were available. This resulted in a reduction in the total sample size for haplogroup A (*n* = 96) comprised of 20 individual shrimp from Aceh, 9 from Cilacap, 15 from Grajagan, 17 from Bali, 15 from Sumbawa, and 20 from Timika, while haplogroup C (*n* = 24) retained the same sample size. Both microsatellite data analyses and mtCR sequence analyses as described above were repeated for the reduced data sets.

## Results

### Microsatellite analysis

DNA from 90 of the 115 *P. monodon* samples from six locations was successfully genotyped at the 15 microsatellite loci targeted. In order for a rigorous comparison of all individuals from all populations, analysis was restricted to only six microsatellite loci for which data were generated across all individual *P. monodon* samples.

Across the 90 genotyped *P. monodon* individuals, allelic polymorphism was present at each of the six microsatellite loci examined, with a total of 38 distinct alleles being identified across all loci (Table2). Locus Pm5271 (21 alleles) displayed the most polymorphism followed by loci Pm528 (20 alleles) and Pm4858 (17 alleles). Locus Pm5625 (8 alleles) displayed the smallest amount of polymorphism. When allelic polymorphism across all six loci was considered collectively, the mean number of alleles (A) per locus ranged from 6.83 in Sumbawa (17 shrimp) to 3.16 in Cilacap (9 shrimp). Within each of the sampled populations, mean observed heterozygosity (*H*_o_) determined across all microsatellites ranged from 0.784 for Aceh (22 shrimp) to 0.532 for Cilacap (9 shrimp) (see Table3). The mean expected heterozygosity (*H*_E_) ranged from 0.425 for Timika to 0.262 for Bali.

The highest allelic mean number (*A*_r_) across all six loci was observed in Sumbawa (3.12) followed by Aceh (3.10) and Timika (3.01) and was lowest for Grajagan (2.67). Most of these were rare alleles, with allele frequencies <5% at each locus, in each population.

Shrimp from Sumbawa possessed the highest (11) private alleles (A_P_), while Cilacap showed the least (2). Inbreeding coefficient (*F*_IS_) calculated for each of the six microsatellite loci for each of the six populations showed statistically significant departure from HWE (*P* < 0.05; Table2). *F*_IS_ was greatest in Timika (0.50) indicating a low level of genetic diversity compared to Cilacap (*F*_IS_ = 0.27) indicating a higher level of genetic diversity.

**Figure 3:**
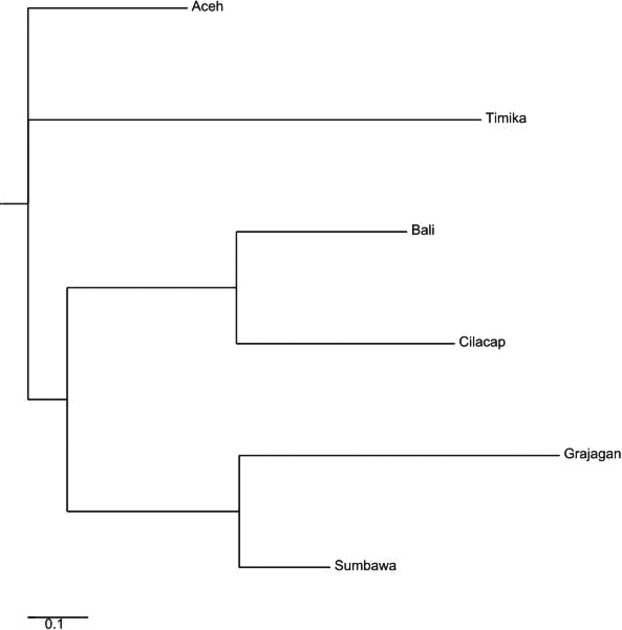
Neighbor-joining phylogenetic tree constructed from microsatellite *F*_ST_ values showing *Penaeus monodon* from six locations in the Indonesian waters clustered within three distinct clades.

The estimated genetic distance among populations (*e F*_ST_ values) revealed statistically significant (*P* < 0.05) differences between all pairs except two population pairs (Table1). The populations for which pairwise *F*_ST_ values were not significant were Aceh–Sumbawa (0.043) and Bali–Cilacap (0.049). In comparison, the highest *F*_ST_ values to other populations were observed for Timika (0.078–0.140) and Grajagan (0.052– 0.140). A neighbor-joining tree generated from the pairwise *F*_ST_ data clustered *P. monodon* from the six locations into three distinct clades, one comprised of shrimp originating only from the Pacific Ocean, another comprised of shrimp from the center of the Indonesian isles, while the third clade comprised of shrimp predominantly from the Indian Ocean. (Fig.3). Clade 1 comprised of one branch of shrimp from Aceh, Clade 2 comprised only of a branch from Timika, and Clade 3 comprised of two sub-branches each split to include shrimp from Bali and Cilacap on one and Grajagan and Sumbawa on the other.

**Table 1:** Pairwise *F*_ST_ among six populations by the use of six microsatellite loci

	Sumbawa	Aceh	Timika	Cilacap	Grajagan	Bali
Sumbawa	–	0.043	0.087	0.115	0.052	0.068
Aceh	–	–	0.078	0.076	0.097	0.073
Timika	–	–	–	0.091	0.140	0.120
Cilacap	–	–	–	–	0.118	0.049
Grajagan	–	–	–	–	–	0.075

**Figure 4:**
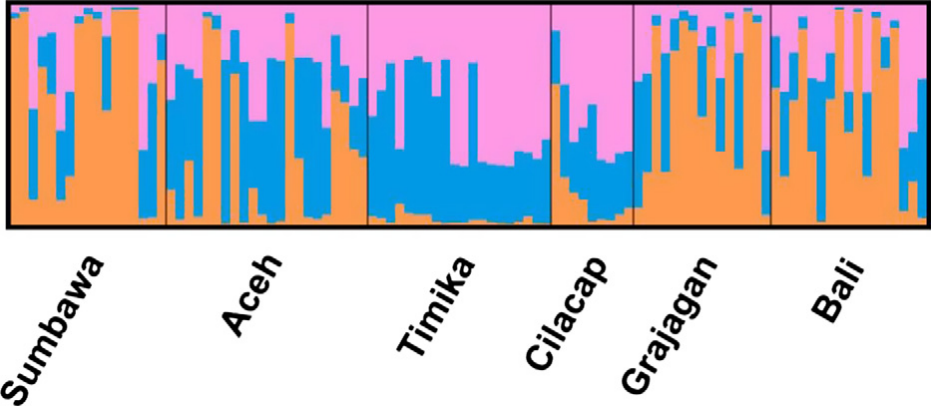
Bayesian analysis of microsatellite genotypes clustered *Penaeus monodon* samples from six locations in the Indonesian waters from three sources of genetic ancestry. Each vertical line pertains to an individual and the proportion of color per individual represents its likely genetic ancestry. Sumbawa, Bali, and Grajagan predominantly from a single source (orange) Timika almost exclusively from the two other sources (blue and pink), while individuals from Aceh and Cilacap showed admixture from all three genetic ancestral sources to varying degrees.

Bayesian analysis of microsatellite allele frequencies suggested that the 90 *P. monodon* samples most likely segregate into three sources of genetic ancestry (Fig.4). Most individuals from Sumbawa, Bali, and Grajagan derived their genetic ancestry from a predominantly single source, and individuals from Timika derived their ancestry almost exclusively from the two other sources, while individuals from Aceh and Cilacap showed admixture from all three ancestral sources to varying degrees.

### mtDNA control region sequence analysis

**Figure 5:**
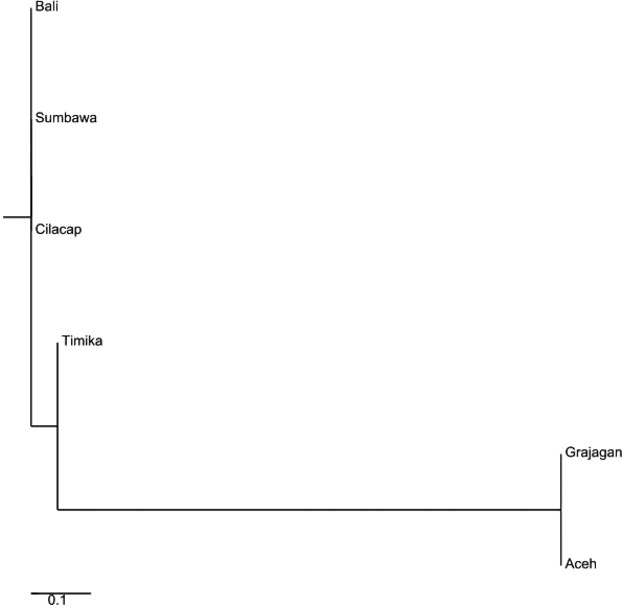
Neighbor-joining phylogenetic tree constructed from haplogroup A mtDNA control region *F*_ST_ values showing *Penaeus monodon* from six locations in the Indonesian waters clustered within four distinct clades.

For haplogroup A, a total of 38 haplotypes were identified in an alignment of a 465-bp mtCR sequence amplified from 111 *P. monodon* which consisted of samples from all six populations. Of these haplotypes, 27 (71%) occurred only once, these haplotypes predominately occurred among shrimp derived from Sumbawa and Grajagan. Both the *F*_ST_ matrix (Fig.5) and the neighbor-joining tree clustered the *P. monodon* individuals into three clades. Clade 1 comprised of shrimp from Bali, Clade 2 from Sumbawa, Clade 3 from Cilacap, and Clade 4 consisted of two branches with shrimp from Grajagan and Aceh forming a branch and Shrimp from Timika forming another branch as an outgroup to the first (Fig.5).

**Figure 6:**
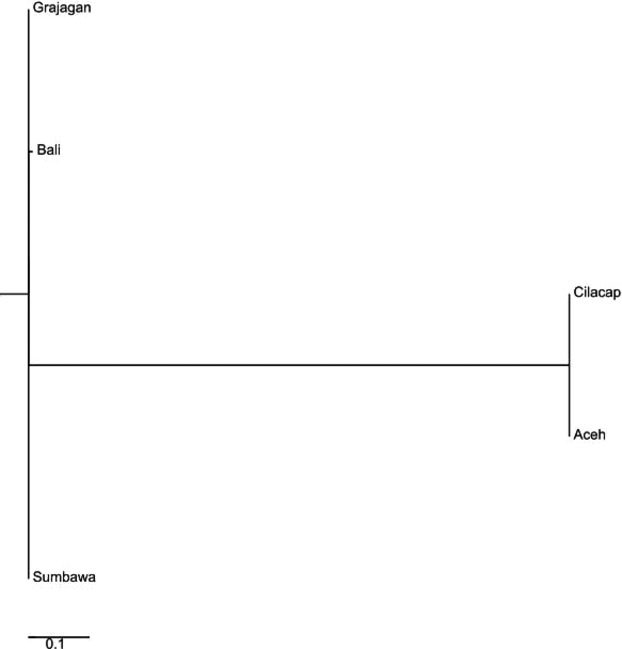
Neighbor-joining phylogenetic tree constructed from haplogroup C mtDNA control region *F*_ST_ values showing *Penaeus monodon* from five locations in the Indonesian waters clustered within four distinct clades.

For haplogroup C, a total of 18 haplotypes were identified in alignments of 465-bp mtCR sequence amplified from 24 *P. monodon* samples. A total of 14 (78%) of these haplotypes occurred only once, which were mostly found among shrimp from Aceh. The *F*_ST_ matrix (Fig.6) and the neighbor-joining tree constructed clustered the 24 *P. monodon* of predefined populations into different clades than that of haplogroup A sequences: Clade 1 comprised of shrimp from Grajagan, Clade 2 those from Bali, Clade 3 clustered shrimp from Cilacap and Aceh together, and Clade 4 comprised only of shrimp from Sumbawa (Fig.6).

### Comparative analysis of microsatellite and mtCR for the elucidation of the probable source of the paralogous sequences

**Figure 7:**
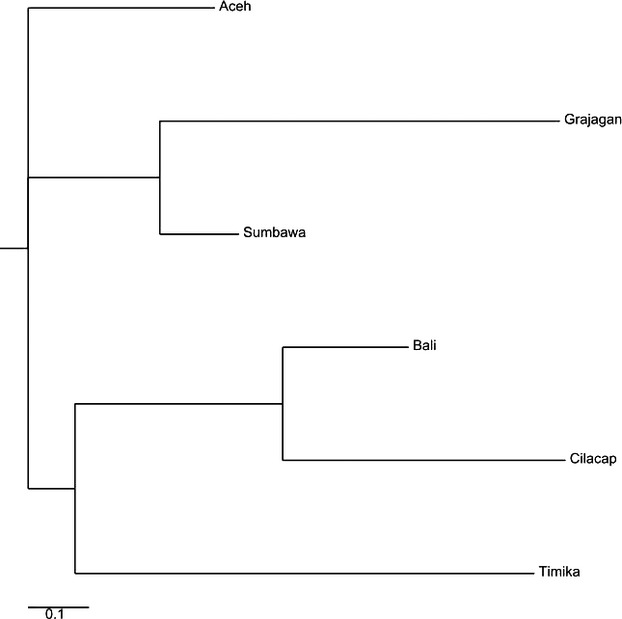
Neighbor-joining phylogenetic tree constructed from reduced haplogroup A data set microsatellite *F*_ST_ values showing *Penaeus monodon* from six locations in Indonesian waters clustered within three distinct clades.

**Figure 8:**
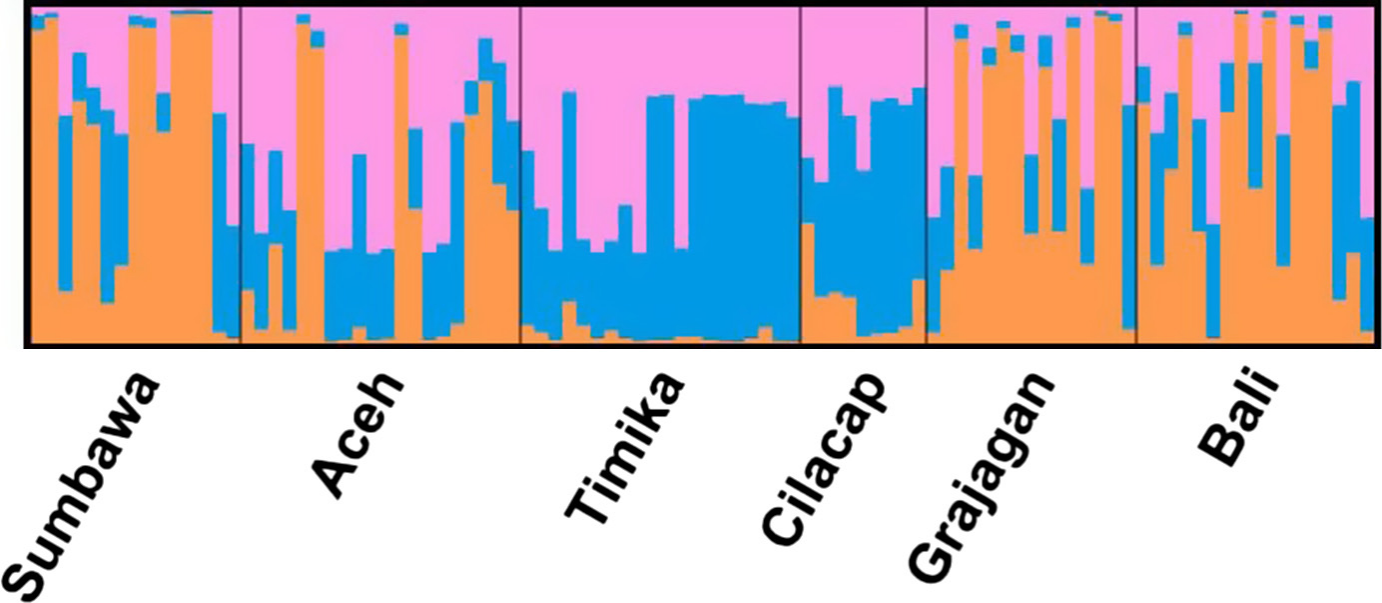
Bayesian analysis of microsatellite genotypes clustered *Penaeus monodon* samples from six locations in the Indonesian waters from three sources of genetic ancestry. Each vertical line pertains to an individual and the proportion of color per individual represents its likely genetic ancestry. Sumbawa, Bali, and Grajagan predominantly from a single source (orange) Timika almost exclusively from the two other sources (blue and pink), while individuals from Aceh and Cilacap showed admixture from all three genetic ancestral sources to varying degrees.

When the *F*_ST_ values from both the microsatellite alleles and the mtCR sequences were calculated for the reduced data set of haplogroup A (96 individuals), the resulting *F*_ST_ matrix (Figs.7 and 8) as well as the neighbor-joining trees generated were then compared.

This neighbor-joining tree generated from the *F*_ST_ calculations of microsatellite DNA clustered the predefined shrimp populations into three clades, Clade 1 comprised of shrimp from Aceh, Clade 2 those from Sumbawa and Grajagan, Clade 3 consisted of two branches; one comprising of Bali and Cilacap and the other with shrimps from Timika which formed a sister branch to the above (Fig.7). This was mostly similar to that of the full data set with the only difference being the clustering of Timika as an outgroup in the third clade as opposed to clustering it as a clade on its own.

In similarity to the full data set, the Bayesian analysis of the microsatellite allele frequencies from the reduced data set for haplogroup A also suggested a likely segregation of the 96 *P. monodon* samples into three sources of genetic ancestry (Fig.8).

**Figure 9:**
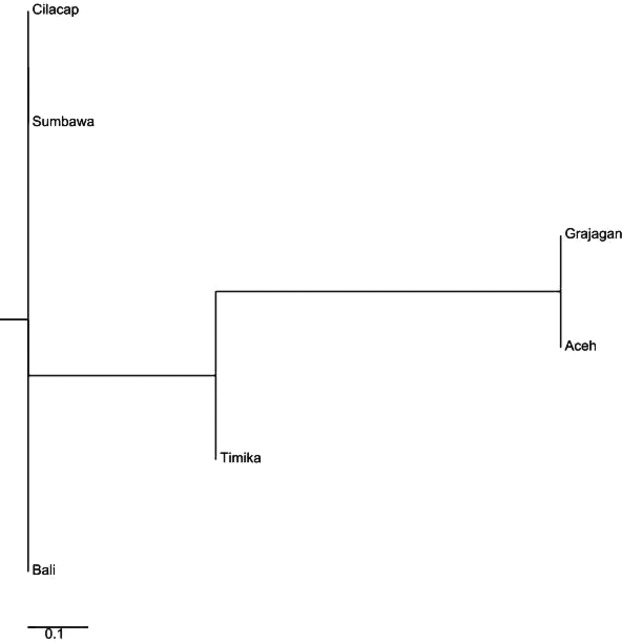
Neighbor-joining phylogenetic tree constructed from reduced haplogroup A data set mtDNA control region *F*_ST_ values showing *Penaeus monodon* from six locations in Indonesian waters clustered within four distinct clades.

The neighbor-joining tree created from mtCR *F*_ST_ values for the reduced data set were completely different from those of the microsatellite data. Its predefined populations were clustered into four clades with Clade 1 comprised of Cilacap, Clade 2 of Sumbawa, Clade 3 branched into two with Timika on one and the other consisted of two nodes with Grajagan and Aceh, and Clade 4 with Bali (Fig.9).

**Figure 10:**
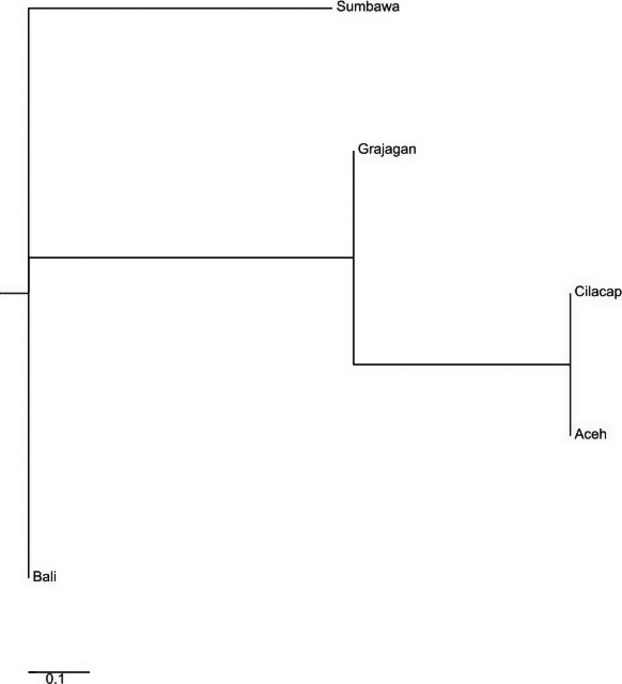
Neighbor-joining phylogenetic tree constructed from reduced haplogroup C data set microsatellite *F*_ST_ values showing *Penaeus monodon* from five locations in the Indonesian waters clustered within three distinct clades.

**Figure 11:**
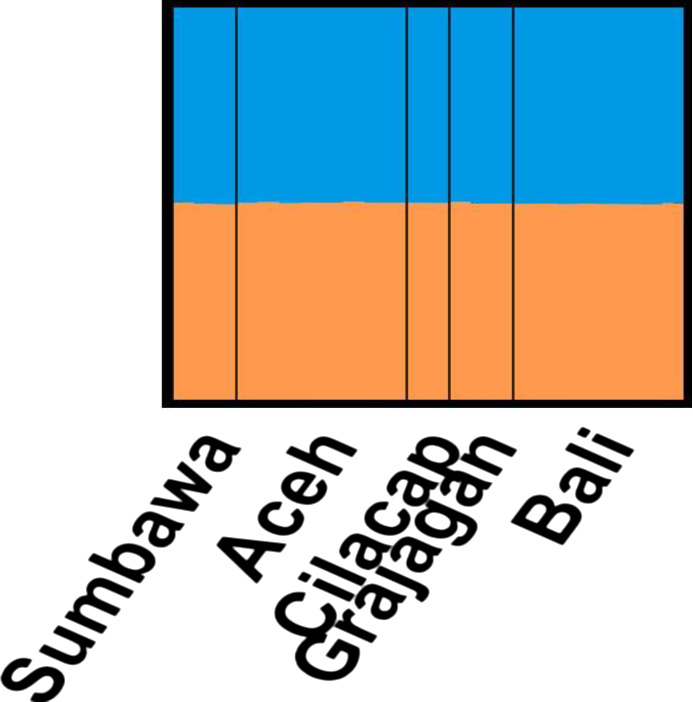
Bayesian analysis of microsatellite alleles from reduced data set from haplogroup C revealed no clear source of genetic ancestry.

For the reduced data set of haplogroup C (24 individuals), *F*_ST_ matrix (Figs.8 and 9) and the subsequent neighbor-joining tree generated from microsatellite data clustered the predefined populations into three Clades as in the full data set. The constituents of the three clades were varied greatly from that of the full data set. The three clades of the reduced data set consisted of Clade 1 with Sumbawa, Clade 2 with two branches, one of which consisted of Grajagan and another with two sub-branches comprised of Cilacap and Aceh, while Clade 3 consisted of only Bali (Fig.10). The Bayesian analysis of the microsatellite DNA data revealed no clear source between these three populations, and this may be due to the small sample size (Fig.11).

**Figure 12:**
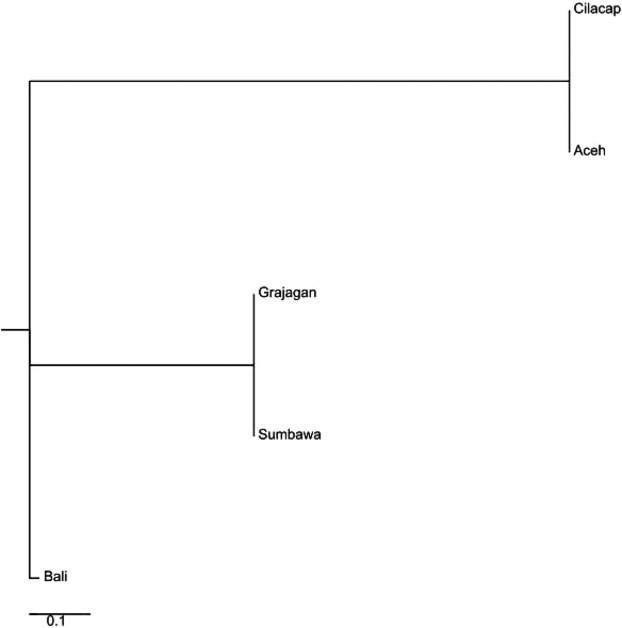
Neighbor-joining phylogenetic tree constructed from reduced haplogroup C mtDNA control region *F*_ST_ values showing *Penaeus monodon* from five locations in Indonesian waters clustered within three distinct clades.

The *F*_ST_ matrix and neighbor-joining tree generated from mtCR data clustered the predefined populations into three clades which, while being very different from those of the reduced data set of haplogroup A and the full data set, were similar to those of haplogroup C reduced microsatellite DNA data set. Clade 1 consisted of two branches, Cilacap and Aceh; Clade 2 with Grajagan and Sumbawa; and Clade 3 of only Bali (Fig.12). Comparatively Clade 3 was seen too similar to that of the microsatellite data, while Clade 1 was seen to closely resemble that of Clade 2 of the microsatellite data.

## Discussion

### Genetic diversity and population structure of *P. monodon* in Indonesian coastal waters

Here we examined the genetic diversity and population structure of 90 specimens of *P. monodon* obtained from six geographic locations. These locations are widely spread across an area of 6000 KM of the coastal regions of the Indonesian islands. Both microsatellite (6 loci) and mtCR sequences were used in the analysis.

### Microsatellite analysis

Based on the microsatellite analysis of the full data set, the genetic diversity among the six *P. monodon* populations was found to be relatively low with respect to the mean number of alleles and heterozygosity values (Table3). This is consistent with a previous analysis based on allozyme data for Indonesian coastal samples (Sugama et al. 2002). Our results also revealed that based on the full data set, *P. monodon* from Aceh and Timika are genetically distinct from each other as well as those from the central Indonesian isles of Cilacap, Grajagan, Sumbawa, and Bali.

Bayesian analysis suggested that the likely genetic ancestry of shrimp from the central Indonesian isles (Sumbawa, Bali, and Grajagan) was different from those from the Pacific Ocean (Timika). This corresponds to findings from other samples from the Indo-Pacific on limited gene flow between the Indian Ocean and the Pacific Ocean (Palumbi 1997; Duda and Palumbi 1999; You et al. 2008). This strong differentiation has been hypothesized to be mostly due to the separation of the Indian and Pacific Ocean populations when lower sea levels restricted the tropical sea passages between these oceans. Another barrier to the movement of *P. monodon* larvae may have been the upsurge of cold water at the base of the Indonesian arc resulting in narrow channels between the eastern Indonesian islands (Palumbi 1997). In addition, this may also explain the high F_IS_ value observed for Timika. Due to the geographic remoteness of Timika in the Pacific Ocean from the rest of the study locations, such genetic distinction was expected. We predict this could be explained with the Member–Vagrant evolutionary model that restricted gene flows result in locally adapted gene pools and stable genetic structures.

### mtDNA control region sequence analysis

*Penaeus monodon* mtCR sequence data for this study was obtained from previously published literature (Walther et al. 2011) where it was found that about a quarter of the sequences were paralogous sequences (haplogroup C), highly diverged from the major haplogroup A. In this publication, we reanalyzed the mitochondrial sequences in conjugation with microsatellite data to elucidate the possible source of the paralogous sequences.

When the data were initially analyzed, the resulting mtCR data for each of the two haplogroups A and C were compared with the microsatellite data. The results of the analysis in terms of the interpopulation structure between the six population groups showed strong differences between microsatellite and mtCR analysis. The analysis of the mtCR sequences for haplogroup A clustered it into four distinct clades, that is, Bali, Sumbawa, Cilacap, and a single clade comprising of Timika and a branch with Grajagan and Aceh. Haplogroup C was also clustered into four distinct clades but differentiated from haplogroup A; with Grajagan, Bali, and Sumbawa forming three distinct clades, and Cilacap and Aceh jointly forming the fourth clade. In the case of haplogroup C, DNA samples from shrimps from Timika were not amplified as this haplogroup represented only the western edge of the Indo-Pacific (Walther et al. 2011).

While no clear conclusion could be drawn from this comparison, it is evident that the analysis was being skewed by data from shrimps for which only microsatellite but no mtCR data was available and vice versa. To control for this bias, the data set was rearranged to include only individuals for which both data types were available.

With the new data set, clearer inferences became possible. While the microsatellite analysis of haplogroup A data set resulted in a different interpopulation structure in comparison with the mtCR analysis, haplogroup C had mostly similar interpopulation structure between the microsatellite analysis and the mtCR analysis. Based on haplogroup A's mtCR analysis being very different from the microsatellite DNA, we hypothesize that haplogroup A is likely comprised of the true mitochondrial sequences which evolved separately from nuclear DNA. In addition, it has been found that phylogenetically, the mitochondrial reference sequence for *P. monodon* (Wilson et al. 2000) belongs to haplogroup A (Walther et al. 2011). Haplogroup C with a comparatively similar interpopulation structure between the microsatellite analysis and the mtCR analysis may point toward it sharing evolutionary history with nuclear DNA. We further hypothesize that haplogroup C may represent Numts (Bensasson et al. 2000, 2001; Frey and Frey 2004; Thalmann et al. 2004). Further evidence is the indication on the basis of phylogenetic trees from (Walther et al. 2011) pointing toward the sequences in haplogroup C being more ancestral than the sequences of haplogroup A.

## Conclusion

Using microsatellite analysis of Indonesian samples, we present further evidence of genetic differentiation between *P. monodon* from the Indian and the Pacific Oceans corroborating earlier reports using older molecular tools (Benzie et al. 2002; Sugama et al. 2002; Waqairatu et al. 2012; You et al. 2008). This variation can provide a basis for a more accurate assignment of *P. monodon* stocks in order to ensure sustainable aquaculture practices as well as the conservation of wild *P. monodon*. Our results also show that the mtCR-like sequences belonging to haplogroup C share evolutionary history with nuclear DNA (Zhang and Hewitt 1996) as would be expected for Numts and hence provide additional evidence that only the mtCR sequences in haplogroup A are authentic mitochondrial sequences. This corroborates previous tentative conclusions regarding the evolutionary source of haplogroup C mtCR sequences in *P. monodon* (Walther et al. 2011) and points to the possibility that there are different migration and dispersal rates for male and female *P. monodon* shrimps in Indonesian coastal waters.

## Figures and Tables

**Figure 13:**
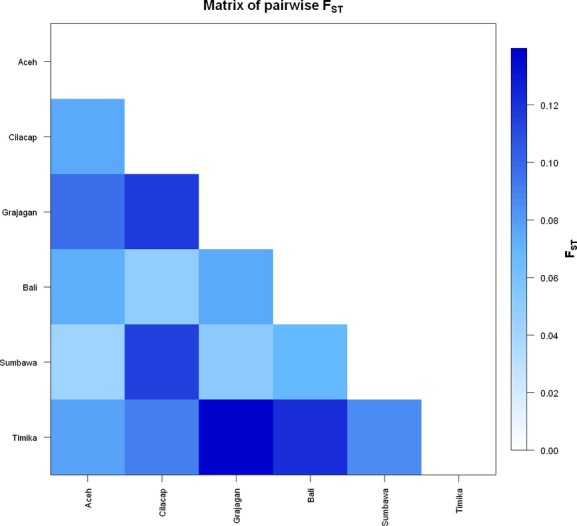
*F*_ST_ matrix of microsatellite data showing *Penaeus monodon* from six locations

**Figure 14:**
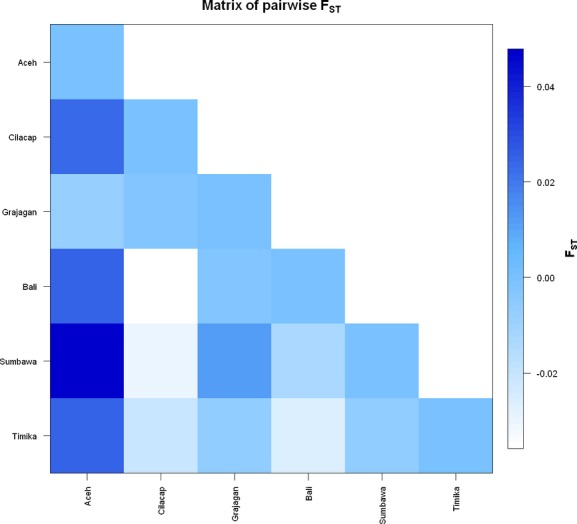
*F*_ST_ matrix of mtDNA control region data from haplogroup A showing *Penaeus monodon* from six locations in the Indonesian waters clustered within four distinct clades.

**Figure 15:**
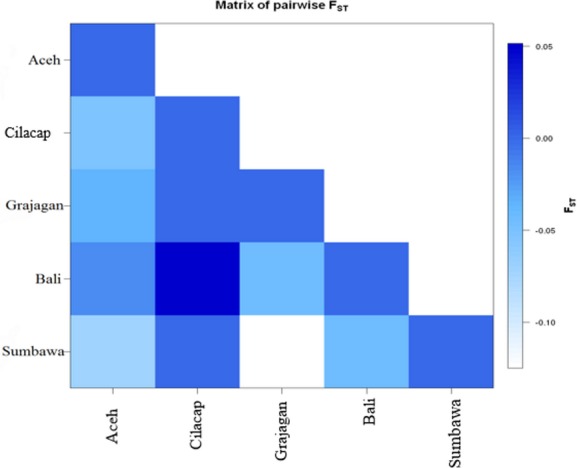
*F*_ST_ matrix of mtDNA control region data from haplogroup C showing *Penaeus monodon* from five locations in the Indonesian waters clustered within four distinct clades.

**Figure 16:**
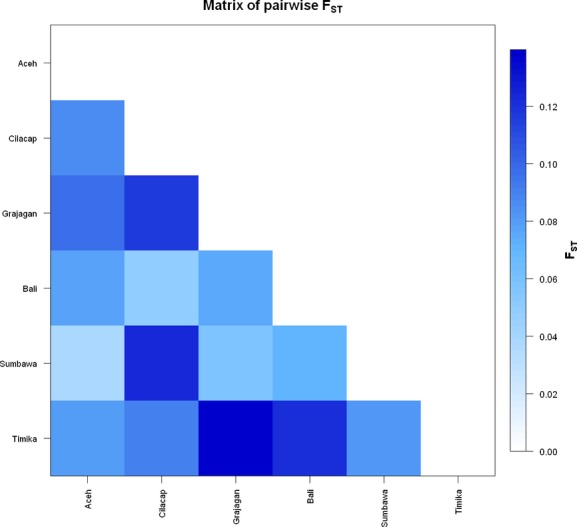
*F*_ST_ matrix of microsatellite data from reduced haplogroup A showing *Penaeus monodon* from six locations in the Indonesian waters clustered within four distinct clades.

**Figure 17:**
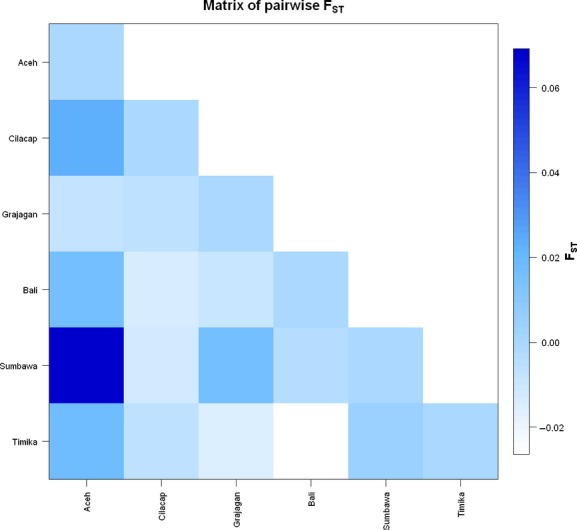
*F*_ST_ matrix of mtDNA control region data from reduced haplogroup A showing *Penaeus monodon* from six locations in the Indonesian waters clustered within four distinct clades.

**Figure 18:**
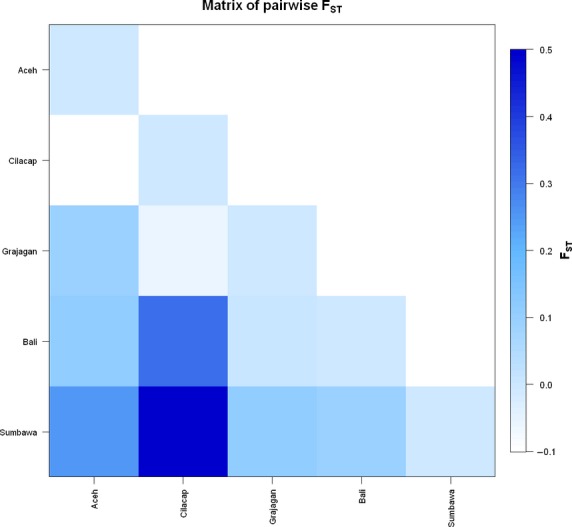
*F*_ST_ matrix of microsatellite data from reduced haplogroup C showing *Penaeus monodon* from five locations in the Indonesian waters clustered within three distinct clades.

**Figure 19:**
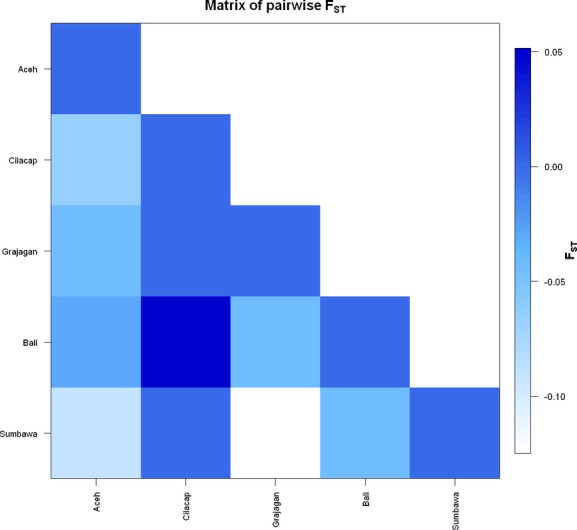
*F*_ST_ matrix of mtDNA control region data from reduced haplogroup C showing *Penaeus monodon* from five locations in the Indonesian waters clustered within three distinct clades.

## Appendix

**A1:** Fifteen polymorphic microsatellite loci which were used in the study (Pan et al. 2004)

No.	Locus	GenBank accession no.	Primers	T. an. (C)
1	PM528^*^	AY500855	F: 5′-GTGTTATTTTCCACGGGTGC	56
			R: 5′-GCTGCAGGAAGTGTAGGGAG	
2	PM580	AY500856	F: 5′-AACTGCCTACAGTGTGTGCG	56
			R: 5′-GAATGGAGCCTGTTGGTTTG	
3	PM1091	AY500857	F: 5′-TTCACGACCCAGTATGTCCA	50
			R: 5′-CAGGTCGCAGGCTCATATTT	
4	PM1713	AY500858	F: 5′-GTTGCGACGGGTTGATTC	60
			R: 5′-TTTATGGCTATGGCTGACAC	
5	PM2345	AY500860	F: 5′-GATATTTCAAGGAATGCTCG	56
			R: 5′-TAATTCGTGCCTTACCTCAT	
6	PM3852	AY500862	F: 5′-TAATGGGCGTAAGTCTTCGG	56
			R: 5′-TGAAAGGAGTCGGGATATGC	
7	PM3854	AY500863	F: 5′-TCTTGGTCGGAATGGGTAAG	56
			R: 5′-TTCTGAGAAGGCACACATGC	
8	PM3945	AY500864	F: 5′-TTTGGACTTCACAATCGGTG	52
			R: 5′-CGGCTGAACAGGTCTGAAAT	
9	PM 4505^*^	AY500867	F: 5′-CTTCTAGCGCCATTTCAAGG	56
			R: 5′-TCCTTCCAGTGTTCGGAGTT	
10	PM4793	AY500868	F: 5′-CATTCACACACGTTCACCTG	56
			R: 5′-CCCAGTTCACGTATCGTGTG	
11	PM4798	AY500869	GTGTGCATACTT	52
			R: 5′-GTTCCCCTCGTGTTTACGAA	
12	PM4858^*^	AY500870	F: 5′-GCCTTGTTACGGTGGAGGTA	56
			R: 5′-CGGCCTATAACTGTCTGCCT	
13	PM4927^*^	AY500872	F: 5′-GGGGAATTAATCTGCCCATT	54
			R: 5′-AATGGCACAAGCAAAAGGAC	
14	PM5271^*^	AY500874	F: 5′-AAAACACTCAGGGGAACACG	53
			R: 5′-CGTGAGCCATAGCTGTAGCA	
15	PM5625^*^	AY500875	F: 5′-AAAAGCCAGAGGAAACGTG	52
			R: 5′-ACAGTGCACGTACCCACAAA	

**A2:** Within-population genetic diversity per microsatellite loci across all six *Penaeus monodon* populations *n*, number of individuals genotyped; *A*, number of alleles; *A*_p_, number of private alleles; *A*_r_, allelic richness; *H*_E_, expected heterozygosity; *H*_o_, observed heterozygosity; *F*_IS_, within-population genetic diversity

Loci	PM 528	PM 505	PM 858	PM 4927	PM 5271	PM 5625	Mean
Allele range (bp)	245–325	333–393	215–295	296–362	222–400	174–246	
Sumbawa (17)							
*n*	17	17	17	17	17	17	
*A*	7	4	7	8	9	6	6.833333
*A*_p_	3	1	2	1	2	2	1.833333
*A*_r_	3.22	3.143	2.595	3.198	3.655	2.963	3.129
*H*_E_	0.589	1.000	1.000	−0.249	0.018	−0.200	0.359
*H*_o_	0.812	0.714	0.630	0.801	0.848	0.714	0.753
*F*_IS_	0.621	1	1	−0.189	0.123	−0.091	0.413
Aceh(22)							
*n*	22	22	22	22	22	22	
*A*	9	6	6	8	13	5	7.833333
*A*_p_	1	1	0	0	4	1	1.166667
*A*_r_	3.318	2.797	2.916	3.066	3.581	2.96	3.106333
*H*_E_	0.404	0.870	0.897	−0.194	0.552	−0.193	0.389
*H*_o_	0.839	0.701	0.745	0.782	0.892	0.745	0.784
*F*_IS_	0.438	0.884	0.906	−0.15	0.577	−0.113	0.427
Timika (20)							
*n*	20	20	20	20	20	20	
*A*	5	6	7	6	10	4	6.333333
*A*_p_	0	1	2	0	3	0	1
*A*_r_	2.618	3.132	2.857	2.833	3.43	3.2	3.011667
*H*_E_	1.000	1.000	0.684	−0.363	0.730	−0.500	0.425
*H*_o_	0.677	0.778	0.729	0.734	0.855	0.677	0.741
*F*_IS_	1	1	0.709	−0.319	0.75	−0.2	0.501
Cilacap (9)							
*n*	9	9	9	9	9	9	
*A*	3	4	3	5	2	2	3.166667
*A*_p_	0	0	1	1	0	0	0.333333
*A*_r_	2.8	4	2.404	3.089	2	1.952	2.7075
*H*_E_	1.000	−0.500	1.000	−0.122	−2.000	−1.250	0.312
*H*_o_	0.600	0.667	0.576	0.742	0.167	0.444	0.532
*F*_IS_	1	0	1	0	0	−1	0.27
Grajagan (15)							
*n*	15	15	15	15	15	15	
*A*	8	2	4	5	5	2	4.333333
*A*_p_	2	0	1	0	0	0	0.5
*A*_r_	3.636	2	2.409	3.233	2.962	1.786	2.671
*H*_E_	0.607	1.000	0.348	−0.324	−0.161	1.000	0.411
*H*_o_	0.848	0.333	0.575	0.756	0.689	0.286	0.581
*F*_IS_	0.667	1	0.432	−0.176	0	1	0.479
Bali (7)							
*n*	7	7	7	7	7	7	
*A*	8	6	5	4	6	2	5.166667
*A*_p_	2	4	2	0	1	0	1.5
*A*_r_	3.478	2.812	2.771	3	3.382	1.952	2.899167
*H*_E_	0.554	0.815	1.000	−0.406	−0.038	−0.350	0.262
*H*_o_	0.842	0.675	0.697	0.711	0.803	0.444	0.695
*F*_IS_	0.604	0.841	1	−0.25	0.074	−0.091	0.384
